# Breast Cancer Phenotype Associated With Li-Fraumeni Syndrome: A Brazilian Cohort Enriched by *TP53* p.R337H Carriers

**DOI:** 10.3389/fonc.2022.836937

**Published:** 2022-03-16

**Authors:** Renata Lazari Sandoval, Natalia Polidorio, Ana Carolina Rathsam Leite, Mariana Cartaxo, Janina Pontes Pisani, Carla Vanessa Quirino, Loureno Cezana, Natálya Gonçalves Pereira, Allan Andresson Lima Pereira, Benedito Mauro Rossi, Maria Isabel Achatz

**Affiliations:** ^1^Oncology Center, Hospital Sírio-Libanês, Brasília, Brazil; ^2^Oncology Center, Hospital Nossa Senhora das Neves, João Pessoa, Brazil; ^3^Oncology Center, Hospital Sírio-Libanês, São Paulo, Brazil; ^4^Oncology Center, Hospital Santa Rita de Cássia, Vitoria, Brazil; ^5^Genetics and Genomics Department, Beneficência Portuguesa, São Paulo, Brazil

**Keywords:** Li-Fraumeni syndrome, *TP53*, R337H, hereditary breast cancer, early-onset breast cancer

## Abstract

Breast cancer (BC) is the most prevalent malignancy in women with Li-Fraumeni syndrome (LFS). The literature on BC in LFS is limited due to its rarity worldwide. A *TP53* founder pathogenic variant (c.1010G>A; p.R337H) is responsible for the higher prevalence of this syndrome among women of Brazilian ancestry.

**Purpose:**

The aim of the study was to describe the BC phenotype expressed by Brazilian female LFS carriers and compare the data between p.R337H and other *TP53* germline pathogenic/likely pathogenic variants (non-p.R337H carriers).

**Methods:**

We searched for cases of *TP53* germline pathogenic/likely pathogenic variant carriers affected by BC included between 2015 and 2020 in the BLiSS (Brazilian Li-Fraumeni Study) registry at the Sírio-Libanês Hospital.

**Results:**

Among 163 adult female carriers from the registry, 91 (56%) had received a BC diagnosis, including 72 p.R337H carriers. BC was the first cancer diagnosed in 90% of cases. Early onset BC (age ≤45 years) was diagnosed in 78.2% of cases (11.5% <31 years; 66.7% 31–45 years; 21.8% >45 years). The median age of BC diagnosis for p.R337H carriers was 39.5 years (range 20–69 years) compared to 34 years (range 21–63 years) for non-p.R337H carriers (p = 0.009). In total, 104 breast tumors were observed in 87 women. Bilateral BC was observed in 29.3% of cases. Histology was available for 96 tumors, comprising 69 invasive breast carcinomas, which were mostly invasive ductal carcinomas (95.6%), 25 ductal *in situ* carcinomas and 2 soft-tissue sarcomas. Overall, 90.5% of invasive breast carcinomas were hormone receptor (HR)-positive, 39.5% were human epidermal growth factor receptor 2 (HER2)-positive, and 32.8% showed HR and HER2 co-expression. In addition, 55.4% of patients opted for contralateral prophylactic mastectomy after a first BC diagnosis. There were no significant differences in the risk of developing contralateral BC or in the immunohistochemical profile between p.R337H and non-p.R337H groups.

**Conclusions:**

The expressed phenotype of p.R337H is similar to that of other *TP53* pathogenic/likely pathogenic variants, except for an average older age at the onset of disease; however, this is still younger than the general population.

## Introduction

Li-Fraumeni syndrome (LFS; OMIM 151623) is an autosomal dominant condition that is associated with an increased risk of developing cancer. It is caused by *TP53* germline pathogenic/likely pathogenic (P/LP) variants. Breast cancer (BC) is the most common cancer diagnosed in women with LFS (1–6). The lifetime risk of BC in women with LFS has been estimated to range 54–85% (7). The risk of BC increases from 20 years of age and peaks at approximately 40 years (7, 8). The median age for the onset of BC is approximately 32–34 years (2, 6, 9).

LFS is highly prevalent in the Southern and Southeastern areas of Brazil, due to the presence of the *TP53* founder variant c.1010G>A (p.Arg337His), also known as variant p.R337H (10, 11). The p.R337H is a missense pathogenic variant (substitution of arginine for histidine at codon 337), located in exon 10, in the oligomerization domain of the protein p53. The frequency of *TP53* p.R337H variant carriers among Brazilian women diagnosed with BC varies from 0.9 to 12%, depending on the geographical region of the studied population and age of BC onset (12–17).

It has been suggested that LFS caused by the *TP53* variant p.R337H has a moderate penetrance when compared to that caused by other *TP53* germline P/LP variants (non-p.R337H) (10, 18, 19). The majority of p.R337H carriers do not fulfill the classic clinical criteria for LFS (20), whereas the frequency of BC in this population is similar to that described in non-p.R337H carriers (10). Nevertheless, age-dependent penetrance seems to be different, less than 20% of p.R337H carriers develop BC before 30 years of age compared to 50% of non-p.R337H carriers, suggesting an age-related penetrance (21).

*TP53* carriers usually have hormone receptor (HR)-positive and human epidermal growth factor receptor 2 (HER2)-positive BCs (22–27). Fitarelli-Kiehl et al. (25) suggested that in p.R337H carriers with BC, there are less HER2-positive tumors. However, there is a lack of available literature that focuses on p.R337H carriers. This information may impact strategic planning for chemoprevention, treatment, and surveillance in this high-risk population.

In this study, we sought to describe the BC phenotype of Brazilian female carriers of LFS and compare the BC features displayed between carriers of the p.R337H and other *TP53* P/LP variants.

## Materials and Methods

### Study Design

All study subjects were participants of the BLiSS (Brazilian Li-Fraumeni Study) registry at the Sírio-Libanês Hospital, Brazil. The eligibility criteria for the current analysis included a confirmed germline P/LP variant in the gene *TP53* and a personal history of BC. Germline testing was performed by commercial laboratories determined by health insurance coverage agreements or out of pocket payment. Cases were ascertained by Sanger sequencing and MLPA (Multiplex Ligation-dependent Probe Amplification), multigene next generation sequencing panels or point mutation sequencing. This study was approved by the Research Ethics Committee of Sírio-Libanês Hospital and the Brazilian Research Ethics Committee (CAAE: 02433418.5.0000.5461). All participants provided informed consent.

Ductal carcinoma *in situ* (DCIS), invasive breast carcinoma (IBC), and breast soft-tissue sarcoma (STS) cases were included. Carriers of more than one P/LP germline variant for an autosomal dominant BC susceptibility gene, possibly mosaic *TP53* P/LP carriers and carriers of TP53 variants with conflicting data of pathogenicity were excluded from the analysis (**Supplementary Figure 1**).

All personal and family history data were ascertained by certified medical geneticists. All clinical and molecular data were de-identified before data sharing and analysis. The collected data included description of the *TP53* germline P/LP variant, age at BC diagnosis, history of unilateral or bilateral BC (synchronous or metachronous), histological subtype and tumor immunohistochemical (IHC) profile, surgical management (lumpectomy vs. mastectomy), contralateral risk reduction mastectomy (CRRM) uptake after a first BC diagnosis, number of primary cancers, and family history of cancer (first, second, or third degree relatives).

All pathology and IHC reports were re-examined. The HR status of the tumor was considered positive if nuclei staining for the estrogen receptor and/or progesterone receptor was greater than or equal to 1%, according to ASCO/CAP guidelines (28). Estrogen receptor (ER) expression of 1–10% was considered ER-low-positive. Additional data based on updated clinical guidelines for BC subtype classification (29) are also available in the supplementary material (**Supplementary Table 1**).

The HER2 expression was recorded using the following scale; 0 for no staining, 1+ for weak cytoplasmic membrane staining in any proportion of tumor cells, 2+ for moderate cytoplasmic membrane staining in at least 10% of tumor cells, and 3+ for intense cytoplasmic membrane staining in at least 30% of tumor cells. Scores of 0 and 1+ were considered negative for HER2 expression. In case of equivocal HER2 expression (score 2+), only samples that had data showing positive detection for HER2 by fluorescent *in situ* hybridization (FISH) were included in the analysis.

### Statistical Analysis

Data were tested for normality using the Kolmogorov–Smirnov and Shapiro–Wilk tests. Values were expressed as medians and percentiles for non-normal continuous variables, and as means and standard deviations for normal continuous variables. Categorical data were presented as absolute values and percentages and were tested using the Pearson χ^2^ and Fisher exact tests, where applicable. Quantitative data were compared by applying Student’s t-test for comparison of the two groups for normally distributed variables and Mann–Whitney U test for non-normally distributed ones. Statistical significance was set at p ≤0.05. Analyses were performed using the SPSS version 21.0 (IBM, NY, USA) software.

## Results

A total of 91 out of 163 (56%) female *TP53* germline P/LP variant carriers had a personal history of BC, and of these, 82 (90%) had BC as the first type of cancer presentation. Furthermore, 72 of the carriers with a personal history of BC harbored the *TP53* p.R337H variant. Other 14 distinct germline *TP53* P/LP variants were identified in the non-p.R337H carriers. To reduce possible bias, carriers of the variants p.D49H, p.R156H, and p.V147A were excluded from the analysis because of conflicting data on pathogenicity (30), which left 15 carriers (17.2%) in the non-p.R337H group (**Supplementary Table 2**). Geographic information about the cohort is detailed in the supplementary material (**Supplementary Table 3**), most of the participants (57/87, 65.5%) lived in the states of São Paulo and Distrito Federal.

The median age at BC diagnosis in the overall cohort was 38 years (range 20–69 years). In addition, 11% (10/87) of the patients were diagnosed under the age of 31, 66.7% (58/87) between 32 and 45 years, and 21.8% above 45 years old. The median age at BC diagnosis in the p.R337H group was 39.5 years (range 20–69 years), which was significantly higher than that observed in the non-p.R337H group (median age 34 years; range 21-63 years; p = 0.009). Furthermore, 43% (31/72) of the p.R337H carriers with BC fulfilled the revised Chompret criteria for LFS (31), in comparison to 60% (9/15) of the non-p.R337H carriers with BC (p = 0.231). All collected data and subgroup analyses are presented in **Table 1**.

**Table 1:** Clinical and pathological characteristics of p.R337H and non-p.R337H carriers.

Cohort characteristics	Total cohort n = 87 (%)	p.R337H n = 72 (%)	Non-p.R337H n = 15 (%)	p-value
**Age of BC diagnosis**				
<31 yr	10 (11.5)	6 (8.3)	4 (26.7)	
31–45 yr	58 (66.7)	49 (68.1)	9 (60)	
>45 yr	19 (21.8)	17 (26.3)	2 (13.3)	
**Median age**	38	39.5	34	0.009
**Revised Chompret criteria**	40 (46)	31 (43.1)	9 (60)	0.231
**Bilateral BC**	12	8	4	0.209
Synchronous	4 (33.3)	2 (25)	2 (50)	
Metachronous	8 (66.6)	6 (75)	2 (50)	
**Histology**				0.015
IBC	69 (72)	60 (77.9)	9 (47.4)	
DCIS	25 (26)	15 (19.5)	10 (52.6)	
STS	2 (2)	2 (2.6)	0	
**HR status**				0.365
HR+	74 (89.2)	61 (91)	13 (81.2)	
HR−	9 (10.8)	6 (9)	3 (18.8)	
**HER2 status**				0.621
HER2+	32 (41)	25 (39.7)	7 (46.7)	
HER2−	46 (59)	38 (60.3)	8 (53.3)	
**HR/HER2 co-expression**				0.219
HR+/HER2+	27 (34.6)	23 (36.5)	4 (26.7)	
HR+/HER2−	43 (55.1)	35 (55.6)	8 (53.3)	
HR−/HER2+	6 (7.7)	3 (4.8)	3 (20)	
HR−/HER2−	2 (2.6)	2 (3.2)	0	

BC, Breast cancer; yr, Years; IBC, Invasive breast carcinoma; DCIS, Ductal carcinoma in situ; STS, Soft tissue sarcoma; IHC, Immunohistochemistry; HR, Hormone receptor; HER2, Human epidermal growth factor receptor 2.

The type of breast surgery was available for 76 patients; 38% of the patients had undergone lumpectomies and 62% had undergone mastectomies. There were 46 patients that had opted for CRRM after a first BC diagnosis, which represented 55.4% of the patients. Cases of synchronous bilateral BC were excluded from the CRRM analysis. Only one patient (1/46, 2.1%) developed BC in the residual breast tissue after prophylactic mastectomy.

There were 17 patients that developed more than one primary BC and the interval between the first and second BC diagnosis ranged from 0 to 21 years. Moreover, 29.3% of the patients (12/41) were affected by bilateral BC, which included four cases of synchronous BC and eight cases of metachronous BC. Cases of CRRM after the first BC diagnosis were excluded from the bilateral breast cancer analysis.

A total of 104 breast tumors were described in the 87 LFS carriers. Pathology reports were available for 96 cases, including 69 IBCs (66 invasive ductal carcinomas and 3 invasive lobular carcinomas), 25 DCISs, and 2 breast STSs. Only 2 patients were diagnosed with invasive lobular carcinomas, out of which, one patient was diagnosed with bilateral synchronous disease at the age of 42. The other patient underwent bilateral mastectomy after a first DCIS diagnosis at the age of 56, but later developed axillary lymph node metastasis from an occult breast carcinoma in the contralateral breast at the age of 63. Both patients were p.R337H carriers.

Among DCIS cases with available HR status, from the total cohort, 85% (17/20) were HR-positive (HR+). The majority of tumors from non-p.R337H carriers were DCIS (p = 0.015). In addition, 90.5% of the total IBCs with available HR status (57/63) were HR+ and 39.5% (24/61) were HER2-positive (HER2+). HR/HER2 co-expression analysis in invasive tumors showed that 57.4% (35/61) were HR+/HER2−, 32.8% were HR+/HER2+, 6.5% were HR−/HER2+, and 3.3% were HR−/HER2−. There were no significant differences found between the p.R337H and non-p.R337H variants groups with regard to HR, HER2 status and HR/HER2 co-expression (**Table 1**). Forty-five IBCs had detailed information about percentage of estrogen receptor expression (>1%, 1–10%, >10%). ER-low-positive IBC represented 6.5% (3/45) of the total IBC samples. According to the St Gallen consensus (29), 33.5% (15/45) of the tumors were luminal A, 31% luminal B-HER2 positive, 20% luminal B-HER2 negative, 13.5% HER2 enriched, 2.2% triple negative (**Supplementary Table 1**).

## Discussion

BC is the most common malignancy in women worldwide. It is the most frequent tumor diagnosed in women with LFS (2–6). LFS carriers have a higher risk of BC, young-onset cancers, multiple primary cancers, and radioinduced tumors (5–8). Therefore, providing adequate screening and follow-up appointments according to the Toronto protocol may enable early cancer detection and reduce cancer morbidity and mortality (32, 33).

Women with LFS should start BC awareness at the age of 18 years and have regular breast MRIs from the age of 20 years or at least 5 years before the earliest diagnosis of BC in the family if this is younger than 20 years of age (34). Risk-reduction mastectomy is an option for primary prevention (35). Early stage disease can be treated with total mastectomy instead of lumpectomy to avoid radiation exposure. Despite these options, there is still limited data available on BCs in LFS patients, especially in relation to p.R337H carriers.

Brazilian LFS carriers are mostly represented by p.R337H carriers, even though other *TP53* germline variants are also found in this population (25, 36, 37). Likewise, although the majority of the LFS carriers in this study harbored the p.R337H variant, 17.2% of the cohort harbored other *TP53* germline P/LP variants. The median age for BC onset in p.R337H carriers was significantly higher (p = 0.009) than the median age of diagnosis in the non-p.R337H group (39.5 versus 34 years, respectively). Similar results have been reported in other studies (9, 23, 25–27, 36). However, it is important to highlight that p.R337H carriers are affected in a lower age than that expected for the general population. Approximately three quarters of our p.R337H cohort with BC were diagnosed before the age of 45 years, and therefore, early high risk screening should be offered to p.R337H carriers.

In this study, almost 60% of the p.R337H group and 40% of the non-p.R337H group failed to fulfill the revised Chompret criteria for LFS. This finding may reflect the suboptimal sensitivity and specificity of LFS clinical criteria (3, 31). A new LFS classification was recently proposed by Kratz et al. (38), which states that *TP53* germline P/LP variant carriers who do not meet the LFS classic or Chompret testing criteria should be defined as attenuated LFS. However, the impact of the new classification in the screening and management of LFS has not yet been determined.

In Brazil, it is estimated that between 0.9 and 12% of women diagnosed with BC carry a germline pathogenic variant of the gene *TP53* (12–17). As clinical criteria are not sufficient to identify all carriers, the cost-effectiveness of universal *TP53* testing in the Brazilian BC population should be evaluated. The same reasoning has previously been applied to pathogenic founder variants detected in the Ashkenazi Jewish population, where *BRCA* universal screening has already been validated (39).

The risk of developing bilateral/contralateral BC ranges between 4 and 70% for LFS female carriers (9, 23, 26, 27, 35, 40). The wide range of this percentage is thought to be due to differences in sample size, study design, and follow-up period between investigations. Furthermore, an association has been made between patient age at the first BC diagnosis and the risk of developing metachronous contralateral BC (40). Bougeard et al. (5) examined the clinical findings recorded from a large cohort of 214 families with LFS, including 415 *TP53* pathogenic variant carriers (non-p.R337H), who were monitored over a 20 year period. The study found that the risk of developing contralateral BC in non-p.R337H LFS carriers was 31%, which is close to the 29.3% risk observed in the p.R337H enriched cohort in the current study.

After the first BC diagnosis, 55% of the cohort opted for CRRM. Therefore, it is proposed that knowledge of *TP53* germline status may influence surgical decision making, although whether genetic testing was performed before or after BC diagnosis was not addressed in the present study. Siegel et al. (35) observed a high uptake (65%) of CRRM in women diagnosed with LFS, particularly in those with an early diagnosis. This corroborates the statistics recorded for CRRM in *BRCA* positive patients (41, 42).

Immunohistochemical features of BC are used routinely to predict prognosis, determine treatment, and plan chemoprevention; and hence, studies incorporating these data are important in the context of LFS. Indeed, it has been shown that HR+ tumors are common in sporadic BC and in BC diagnosed in LFS carriers (9, 22, 23, 26, 27, 43); and the results from this study are in accordance with those of previous studies. It is possible that chemoprevention through hormonal therapy may play a role in preventing BC in LFS carriers, and future studies should evaluate this as a potential preventive treatment. Treatment-oriented classification of subgroups of BC has evolved in the last decade (28, 29, 44, 45). Previous published LFS BC studies considered ER-positive cut off greater than or equal to 1% (9, 25–27), nevertheless, updated guidelines (28, 29) incorporated the concept of ER-low-positive due to its potential impact in prognosis and predictive role in neoadjuvant chemotherapy response (44). Our study observed 6.5% (3/45) of ER-low-positive IBCs. The retrospective design of our study imposed some limitations regarding missing/not available data. The frequency of ER-low-positive tumors should be systematically assessed in prospective LFS BC studies.

HER2+ tumors are associated with higher mortality (43), and *TP53* P/LP variant carriers are more likely to have HER2+ breast tumors (9, 22–24, 26, 27). According to some studies, BCs that are found in p.R337H carriers are less likely to be HER2+ than those detected in non-R337H *TP53* P/LP variant carriers (24, 25). It can be noted that although Fortuno et al. (24) observed a lower proportion of HER2+ breast tumors in p.R337H carriers when compared to non-R337H *TP53* P/LP variant carriers, this difference was only significant for the age group of 40–49 years. Furthermore, another study that reported similar findings had included BC samples with equivocal HER2 amplification that had not been confirmed by FISH analysis in the test set (25). The current study showed that 41.5% of IBCs from p.R337H carriers were HER2+. Special attention should be made when comparing LFS BC published data in relation to HER2 status. The clinical relevance of HER2 positivity is well established for IBC (45). The significance of HER2 overexpression in DCIS is not well defined (46). Packwood et al. (26) and Kuba et al. (27) have included IBCs and DCISs to calculate overall HER2 positivity. Other studies used the term ‘breast tumors’ but were not clear about the inclusion of IBCs and/or DCISs (24, 25).

In the present study there was a recruitment bias. The majority of the participants were carriers of the founder pathogenic variant p.R337H and lived in the Southeast and Central-West of the country (**Supplementary Table 3**). Although this founder variant was initially found to be present in the Brazilian population from the South and Southeastern areas, the existence of p.R337H variant carriers in the Central-West of the country suggests that internal migrations may be responsible for the spread of the founder variant through different regions of Brazil.

Although the present study included the largest BC cohort of p.R337H carriers to date (n = 72), the retrospective design of the study imposed a few limitations. It would be beneficial to establish a Brazilian prospective cohort with cancer affected and unaffected LFS carriers in order to identify potential risk modifiers of BC, such as environmental exposures, breast feeding, previous cancer treatment, and family history of BC. In addition, it would be useful to examine the consequences of the *TP53* germline status on oncological treatment approaches and the effects of intensive surveillance on morbidity. The Brazilian population offers a unique opportunity for a better understanding of LFS in the context of *TP53* p.R337H variant carriers. Therefore, Brazilian academic and medical institutions should add efforts for the creation of a National LFS Registry.

Finally, female carriers of the LFS *TP53* p.R337H variant have a high risk of BC and bilateral disease. The p.R337H variant has a similar phenotype to *TP53* non-R337H P/LP variants, except for a slightly older onset age for BC (which is still considered a young age for BC onset). For these reasons, carriers of the p.R337H variant should follow the same management guidelines as *TP53* non-R337H P/LP carriers.

## Data Availability Statement

The original contributions presented in the study are included in the article/**Supplementary Material**, further inquiries can be directed to the corresponding author.

## Ethics Statement

The studies involving human participants were reviewed and approved by the Ethics Committee of Hospital Sírio-Libanês and the Brazilian Research Ethics Committee (CAAE: 02433418.5.0000.5461). The patients/participants provided their written informed consent to participate in this study.

## Author Contributions

Concept and Design: RLS. Acquisition, analysis, or interpretation of data: RLS, NP, MC, JPP, CVQ, ACRL, LC, NGP, AALP, BMR, and MIA. Drafting of the manuscript: RLS and MIA. Critical revision of the manuscript for important intellectual content: RLS, NP, MC, JPP, CVQ, ACRL, LC, NGP, AALP, BMR, and MIA. Supervision: RLS and MIA. All authors listed have made a substantial, direct, and intellectual contribution to the work and approved it for publication.

## Funding

We received financial support from the Stand Up to Cancer (SU2C) for this study.

## Conflict of Interest

The authors declare that the research was conducted in the absence of any commercial or financial relationships that could be construed as a potential conflict of interest.

## Publisher’s Note

All claims expressed in this article are solely those of the authors and do not necessarily represent those of their affiliated organizations, or those of the publisher, the editors and the reviewers. Any product that may be evaluated in this article, or claim that may be made by its manufacturer, is not guaranteed or endorsed by the publisher.
