# Supplementary material for: Breast Cancer Phenotype Associated With Li-Fraumeni Syndrome: A Brazilian Cohort Enriched by TP53 p.R337H Carriers
Source: Front Oncol. 2022 Mar 16;12:836937. doi: 10.3389/fonc.2022.836937 (PMC8966034; doi:10.3389/fonc.2022.836937)
Supplement: Supplementary file 1 [file DataSheet_1.docx]

Supplementary Material

# Supplementary Figures


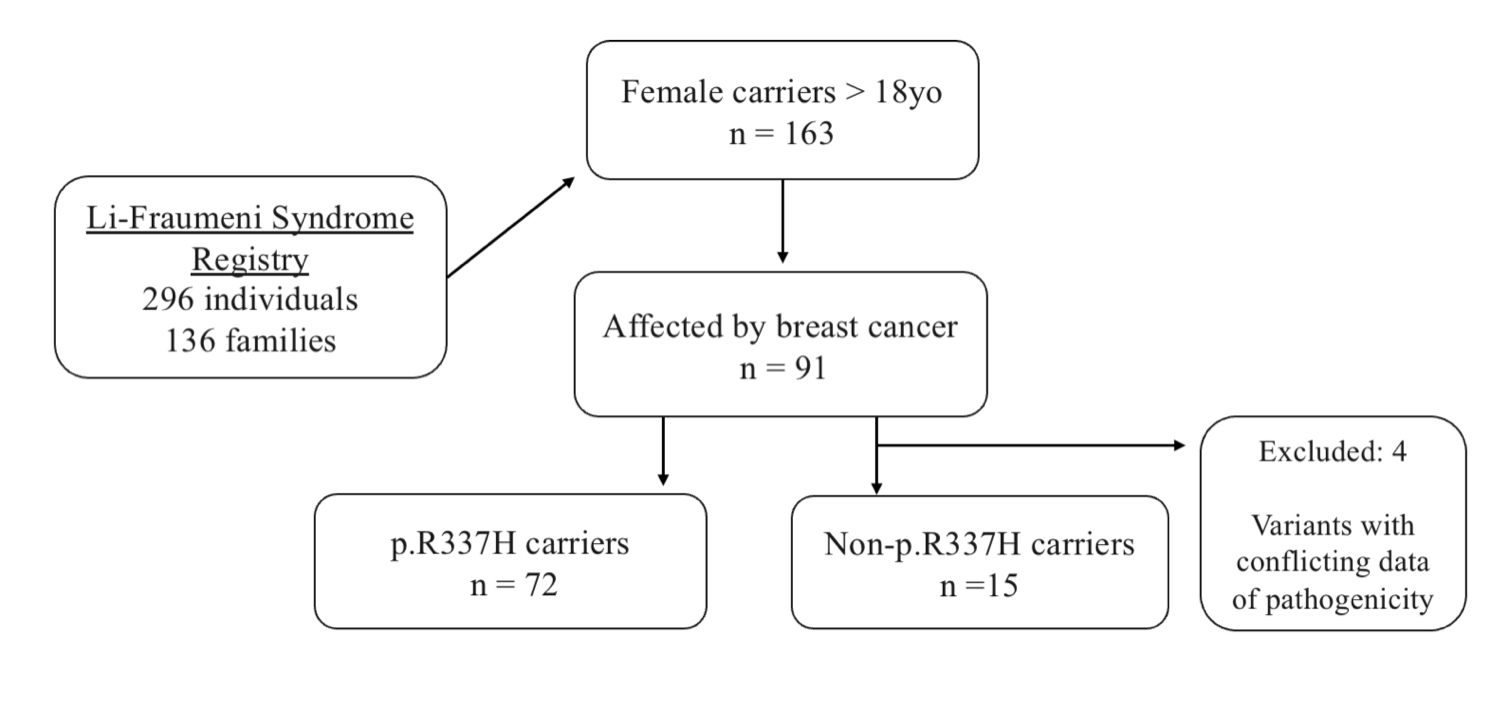


**Figure 1.** Cohort selection pipeline.

# Supplementary Tables

**Suplementary Table 1.** Breast tumor data according to the St Gallen International Expert Consensus (2015).

| Breast cancer subtype | Total n^o^ of IBC, n=69 | Total n^o^ of IBC in p.R337H carriers, n=60 | Total no of IBC in non-p.R337H carriers, n=9 |
| --- | --- | --- | --- |
| Luminal A | 15 (33.5%) | 13 | 2 |
| Luminal B-HER2 negative | 9 (20%) | 8 | 1 |
| Luminal B-HER2 positive | 14 (31%) | 13 | 1 |
| HER2-enriched | 6 (13.5%) | 4 | 2 |
| Triple negative | 1 (2%) | 1 | 0 |
| Missing data for St Gallen classification* | 24 | 21 | 3 |

Abbreviations: Invasive breast cancer (IBC).

**Supplementary Table 2.** *TP53* germline P/LP variants included in the study.

| c.DNA | Protein change | dbSNP | ClinVar classification | No. of patients |
| --- | --- | --- | --- | --- |
| c.829T>C | p.R273H | [rs28934576](https://www.ncbi.nlm.nih.gov/snp/rs28934576) | P | 2 |
| c.916C>T | p.R306* | [rs121913344](https://www.ncbi.nlm.nih.gov/snp/rs121913344) | P | 1 |
| c.733G>A | p.G245S | [rs28934575](https://www.ncbi.nlm.nih.gov/snp/rs28934575) | P | 2 |
| c.375G>A | p.T125T | [rs55863639](https://www.ncbi.nlm.nih.gov/snp/rs55863639) | P | 1 |
| c.743G>A | p.R248Q | [rs11540652](https://www.ncbi.nlm.nih.gov/snp/rs11540652) | P | 3 |
| c.829T>C | p.C277R | [rs1064795369](https://www.ncbi.nlm.nih.gov/snp/rs1064795369) | LP | 1 |
| c.741_742delinsTT | p.R248W | [rs1555525498](https://www.ncbi.nlm.nih.gov/snp/rs1555525498) | LP | 1 |
| c.219_220dupGG | p.A74Gfs*50 | - | LP | 1 |
| c.451C>G | p.P151A | [rs28934874](https://www.ncbi.nlm.nih.gov/snp/rs28934874) | P | 1 |
| c.1052delA | p.K351Rfs* | - | LP | 1 |
| Del exons 2–10 | - | - | LP | 1 |

Abbreviations: Pathogenic (P); Likely pathogenic (LP).

**Supplementary Table 3.** Brazilian geographic regions and the states of residency of the studied cohort.

| Geographic regions | Women with LFS  > 18 years-old from BLiSS  (N^o^ p.R337H carriers) | Women with LFS affected by breast cancer  (N^o^ p.R337H carriers) |
| --- | --- | --- |
| North | 0 | 0 |
| Northeast  Alagoas  Bahia  Ceará | 1 (1)  1 (1)  2 (0) | 0  1 (1)  0 |
| Central-West  Distrito Federal  Goiás | 25 (23)  8 (8) | 13 (11)  5 (5) |
| Southeast  Espírito Santo  Minas Gerais  Rio de Janeiro  São Paulo | 6 (5)  15 (14)  2 (1)  86 (75) | 4 (3)  10 (9)  1 (0)  44 (36) |
| South  Rio Grande do Sul  Santa Catarina  Paraná | 1 (0)  2 (2)  12 (11) | 1 (0)  1 (1)  6 (5) |
| Missing data | 2 (1) | 1 (1) |
| Total | 163 (142) | 87 (72) |

**
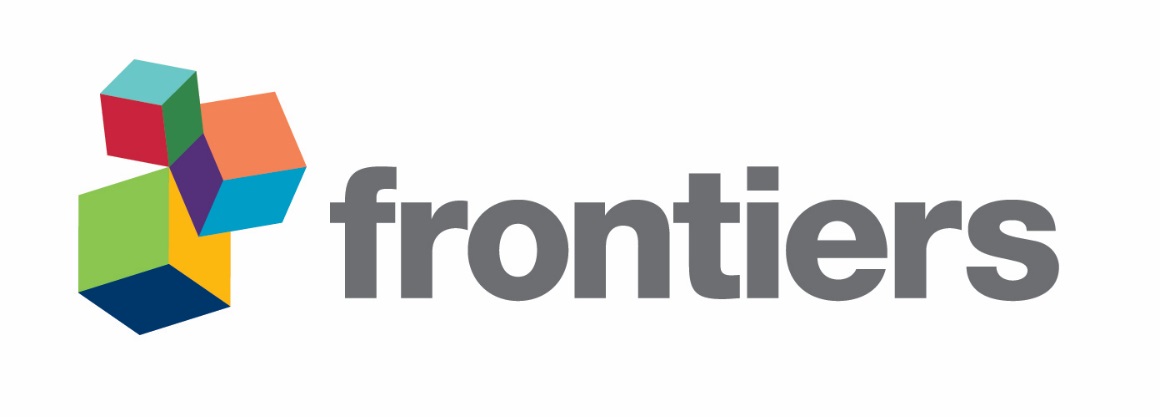
**
